# Supplementary material for: Identification of long regulatory elements in the genome of Plasmodium falciparum and other eukaryotes
Source: PLoS Comput Biol. 2021 Apr 16;17(4):e1008909. doi: 10.1371/journal.pcbi.1008909 (PMC8081344; doi:10.1371/journal.pcbi.1008909)
Supplement: S1 Fig — (PDF) [file pcbi.1008909.s001.pdf]

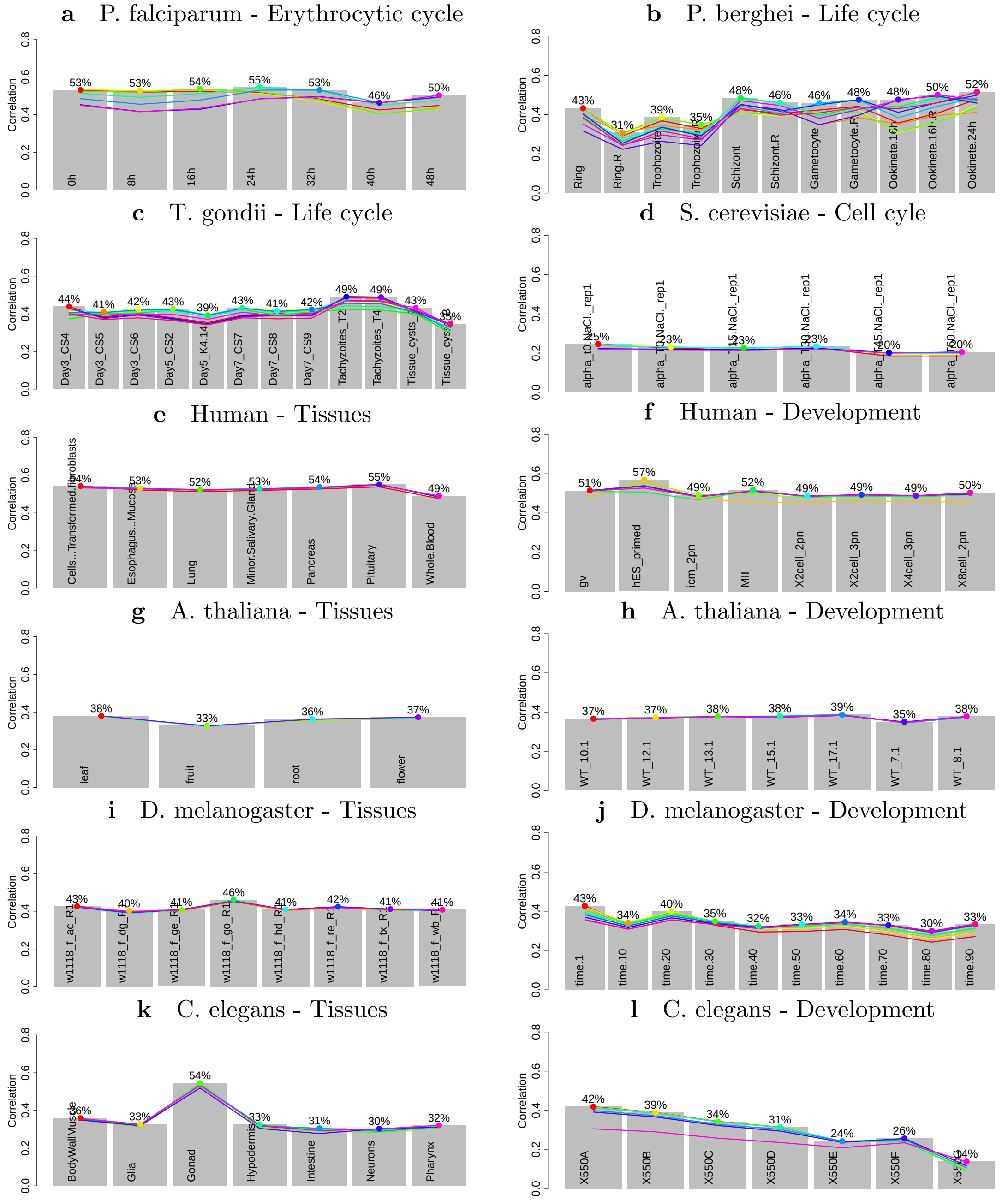

Figure S1: **Accuracy of DExTER models trained using only di-nucleotides on whole region.** These histograms report the accuracy of models that use as predictive variables only the frequencies of the 16 dinucleotides computed on the whole sequences.
