# Supplementary material for: Identification of long regulatory elements in the genome of Plasmodium falciparum and other eukaryotes
Source: PLoS Comput Biol. 2021 Apr 16;17(4):e1008909. doi: 10.1371/journal.pcbi.1008909 (PMC8081344; doi:10.1371/journal.pcbi.1008909)
Supplement: S2 Fig — (PDF) [file pcbi.1008909.s002.pdf]

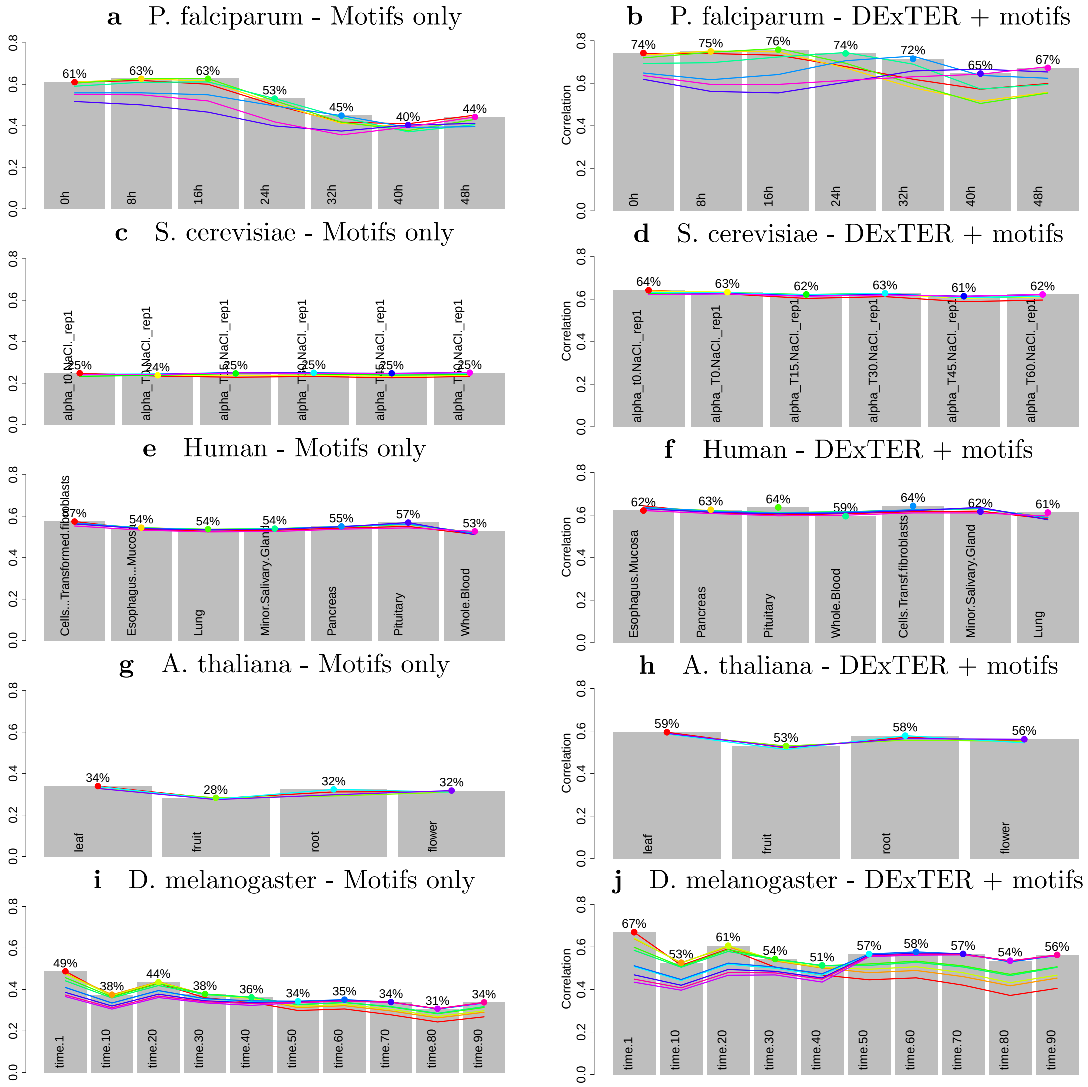

Figure S2: **Accuracy of models trained using TF motifs, with or without addition of DExTER variables.** Histograms on the left-hand side (a, c, e, g i) reports the accuracy of models that only use motif scores as predictive variables. JASPAR 2020 motifs were used for these analyses, except for *P. falciparum* for which we used the 23 ApiAP2 PWMs identified in reference [?]. For JASPAR motifs, we used the non-redundant core collections from Vertebrate, Plants, Insects, and Fungi for Human, *A. thaliana*, *Drosophila*, and yeast, respectively. Histograms on the right-hand side (b, d, f, h, j) report the accuracy achieved by models that use both these motif scores and the DExTER variables of Figure 2.
