# Supplementary material for: Identification of long regulatory elements in the genome of Plasmodium falciparum and other eukaryotes
Source: PLoS Comput Biol. 2021 Apr 16;17(4):e1008909. doi: 10.1371/journal.pcbi.1008909 (PMC8081344; doi:10.1371/journal.pcbi.1008909)
Supplement: S3 Fig — (PDF) [file pcbi.1008909.s003.pdf]

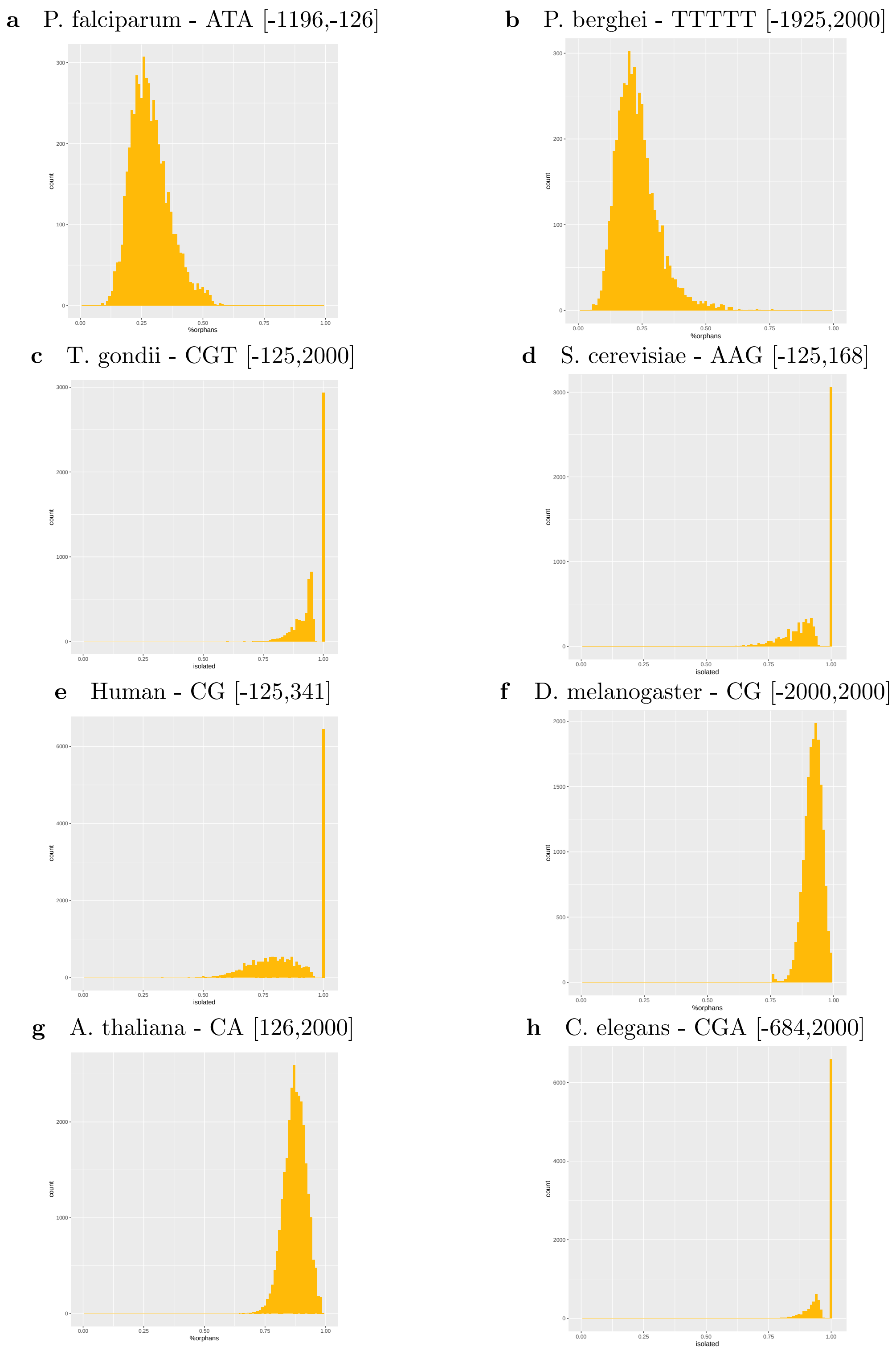

Figure S3: **Proportion of isolated occurrences of identified k-mers in regions.** These histograms report the proportion of isolated occurrences found in each gene of each species, for the most important variable of each species. A k-mer occurrence is considered as isolated if it is not immediately followed or preceded by another occurrence of the same k-mer. For these analyzes, k-mer repetitions can be either immediately consecutive or overlapping (for example, k-mer ATA is considered as being non-isolated in the two sequences ATAATA and ATATA).
