# Supplementary material for: Identification of long regulatory elements in the genome of Plasmodium falciparum and other eukaryotes
Source: PLoS Comput Biol. 2021 Apr 16;17(4):e1008909. doi: 10.1371/journal.pcbi.1008909 (PMC8081344; doi:10.1371/journal.pcbi.1008909)
Supplement: S4 Fig — (PDF) [file pcbi.1008909.s004.pdf]

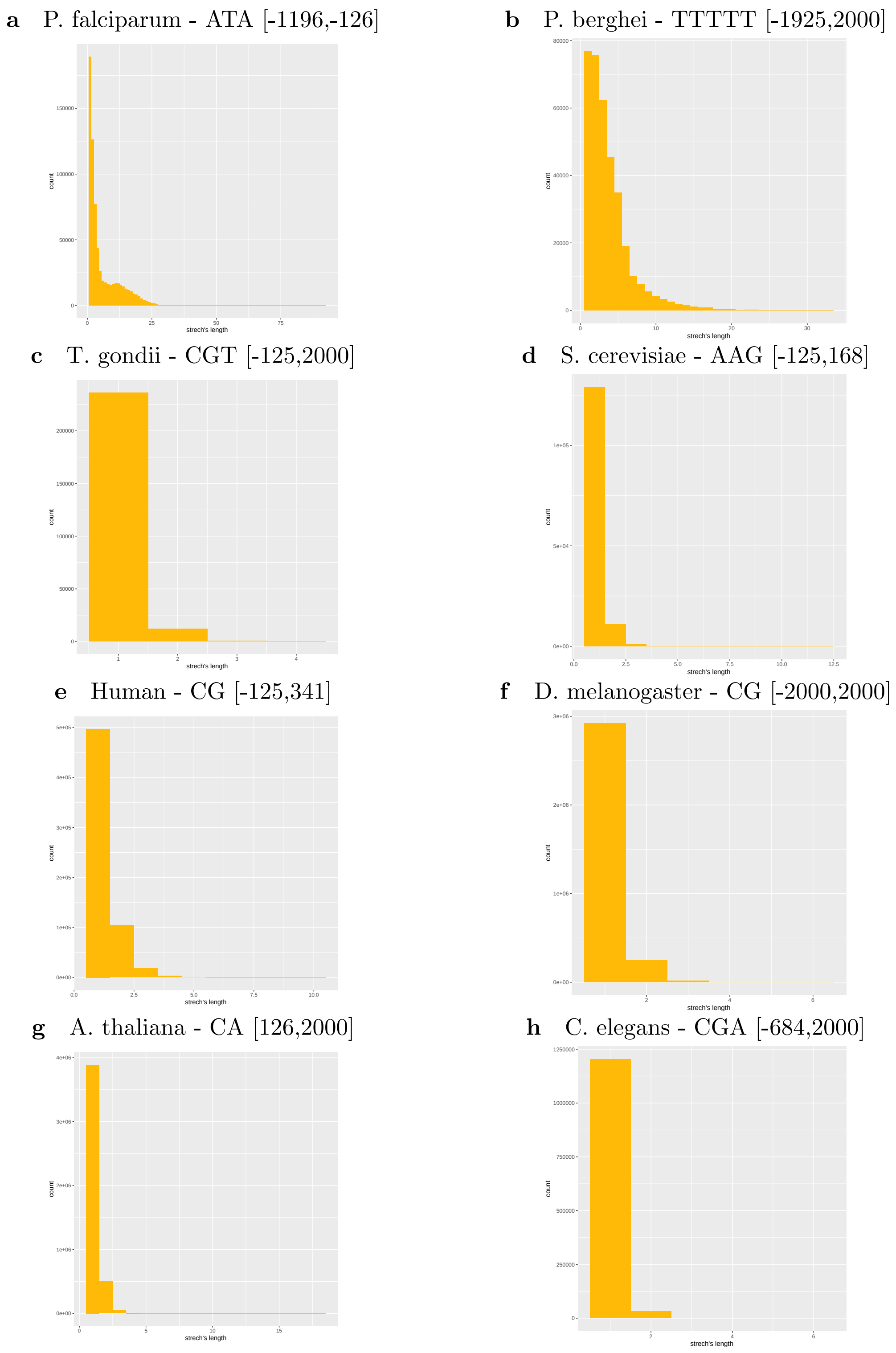

Figure S4: **Length of repetitive blocks.** These histograms report the length of the repetitive blocks in which the different k-mer occurrences are found. In this figure, isolated occurrences appears in "blocks" of length 1. As in Supp. Figure 3, k-mer repetitions can be either immediately consecutive or overlapping (for example, ATAATA and ATATA are tow repetitive blocks made up of two ATA occurrences each). Note that this figure report the length of the blocks in which each k-mer occurrence belong. This means that the same block appear as many times as the number of occurrences it contains.
