# Supplementary material for: Identification of long regulatory elements in the genome of Plasmodium falciparum and other eukaryotes
Source: PLoS Comput Biol. 2021 Apr 16;17(4):e1008909. doi: 10.1371/journal.pcbi.1008909 (PMC8081344; doi:10.1371/journal.pcbi.1008909)
Supplement: S6 Fig — (PDF) [file pcbi.1008909.s006.pdf]

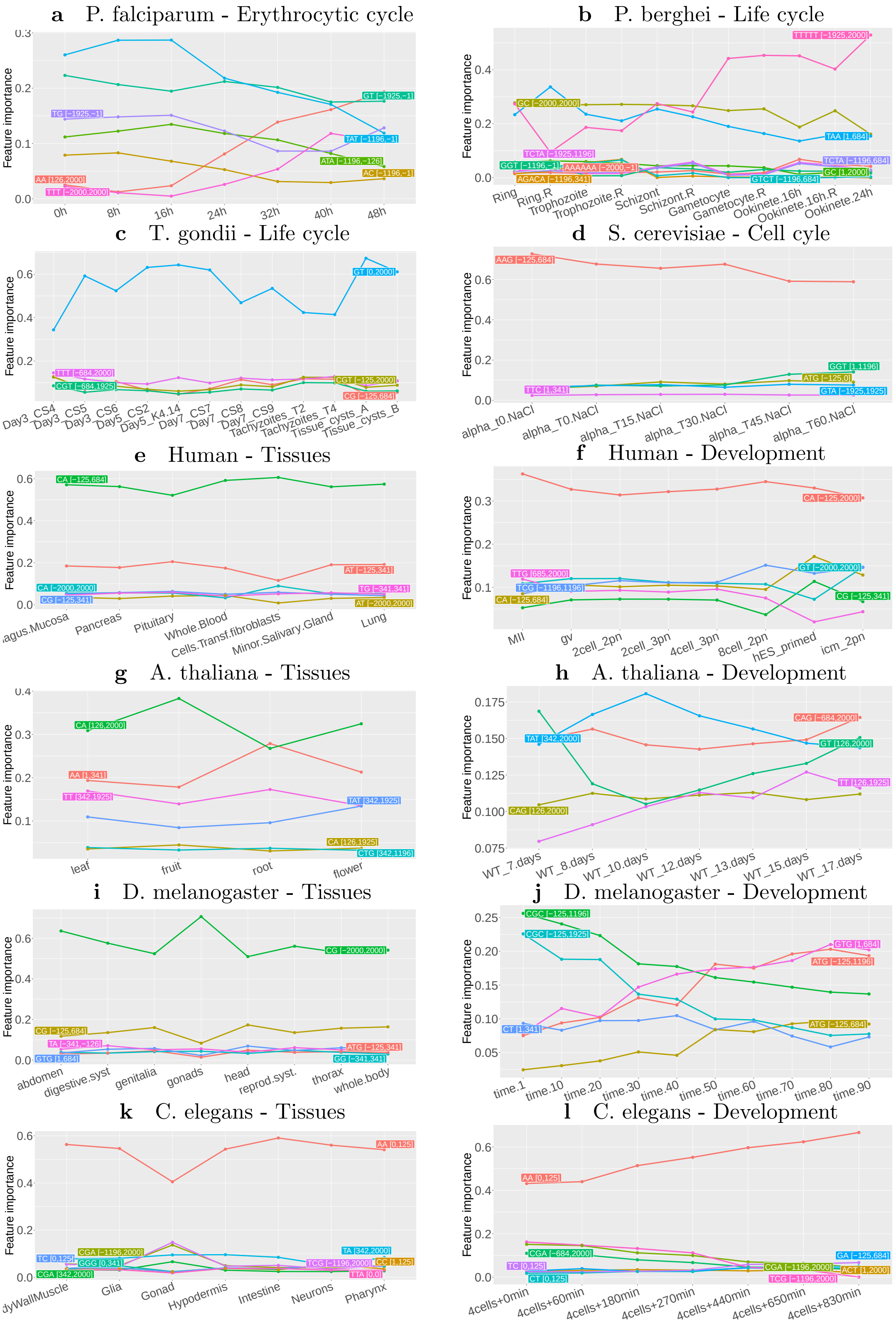

Figure S6: **Variable importance in the different models.** For each expression series, the 5 most important variables of each condition were identified, and their importance values were computed for all conditions of the series.
