# Supplementary material for: Identification of long regulatory elements in the genome of Plasmodium falciparum and other eukaryotes
Source: PLoS Comput Biol. 2021 Apr 16;17(4):e1008909. doi: 10.1371/journal.pcbi.1008909 (PMC8081344; doi:10.1371/journal.pcbi.1008909)
Supplement: S7 Fig — (PDF) [file pcbi.1008909.s007.pdf]

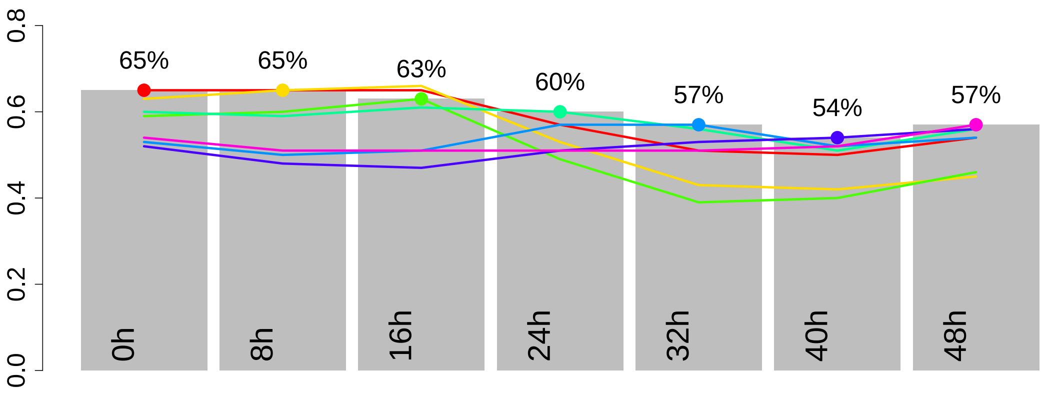

Figure S7: **Prediction of *P. falciparum* gene expression during erythrocytic cycle, with convolutional neural networks.** Grey charts represent the accuracy, measured as the correlation between predicted and observed gene expression, of the CNNs learned on different time points. Colored curves summarize the accuracy of a CNN learned on a specific time point (identified by a big dot of the same color) when used to predict the other time points of the erythrocytic cycle.
