# Supplementary figures and images for: Identification of long regulatory elements in the genome of Plasmodium falciparum and other eukaryotes
Source: PLoS Comput Biol. 2021 Apr 16;17(4):e1008909. doi: 10.1371/journal.pcbi.1008909 (PMC8081344; doi:10.1371/journal.pcbi.1008909)

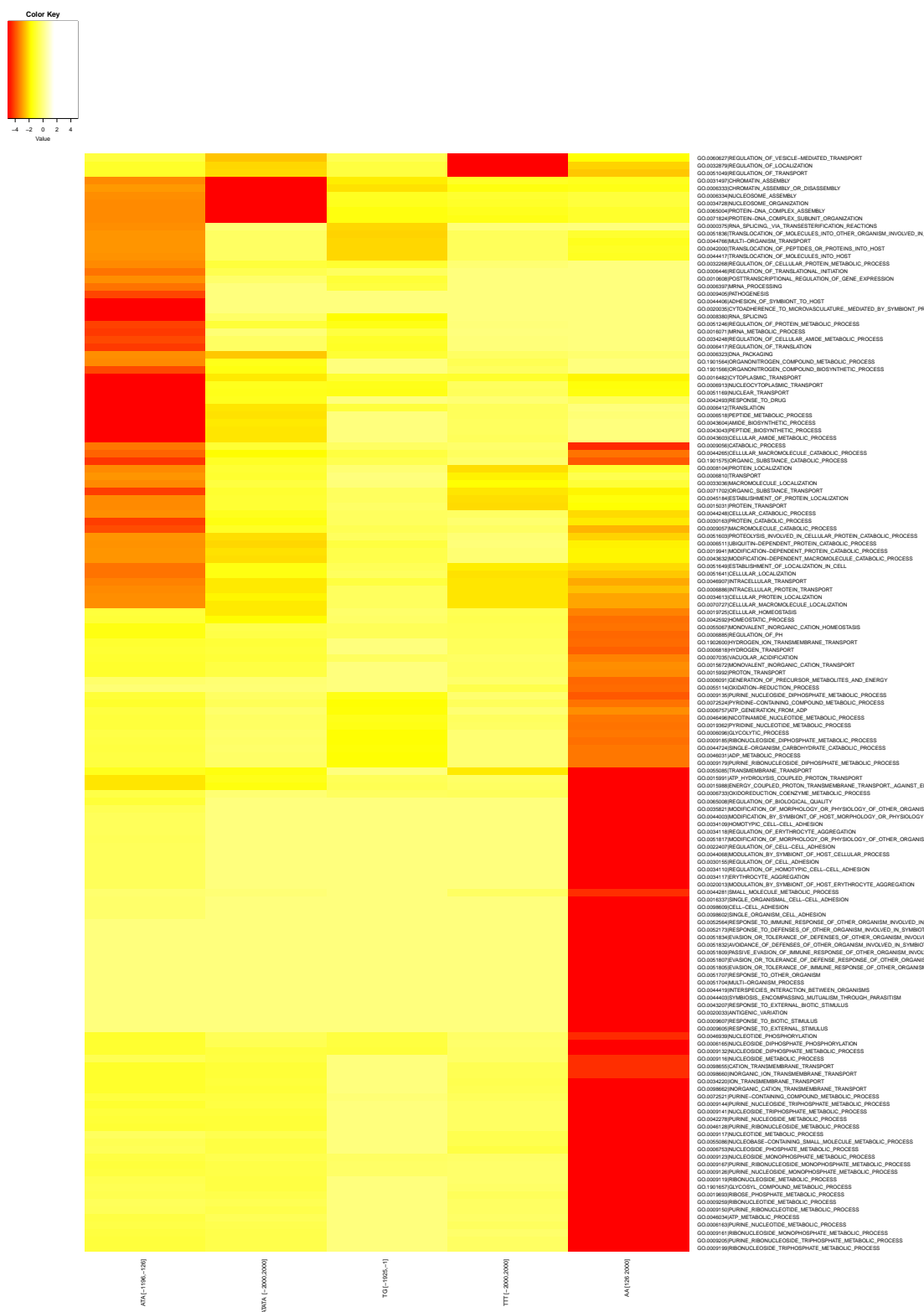

Supplement: S8 Fig — (PDF) [file pcbi.1008909.s008.pdf]
