# Supplementary material for: Identification of long regulatory elements in the genome of Plasmodium falciparum and other eukaryotes
Source: PLoS Comput Biol. 2021 Apr 16;17(4):e1008909. doi: 10.1371/journal.pcbi.1008909 (PMC8081344; doi:10.1371/journal.pcbi.1008909)
Supplement: S9 Fig — (PDF) [file pcbi.1008909.s009.pdf]

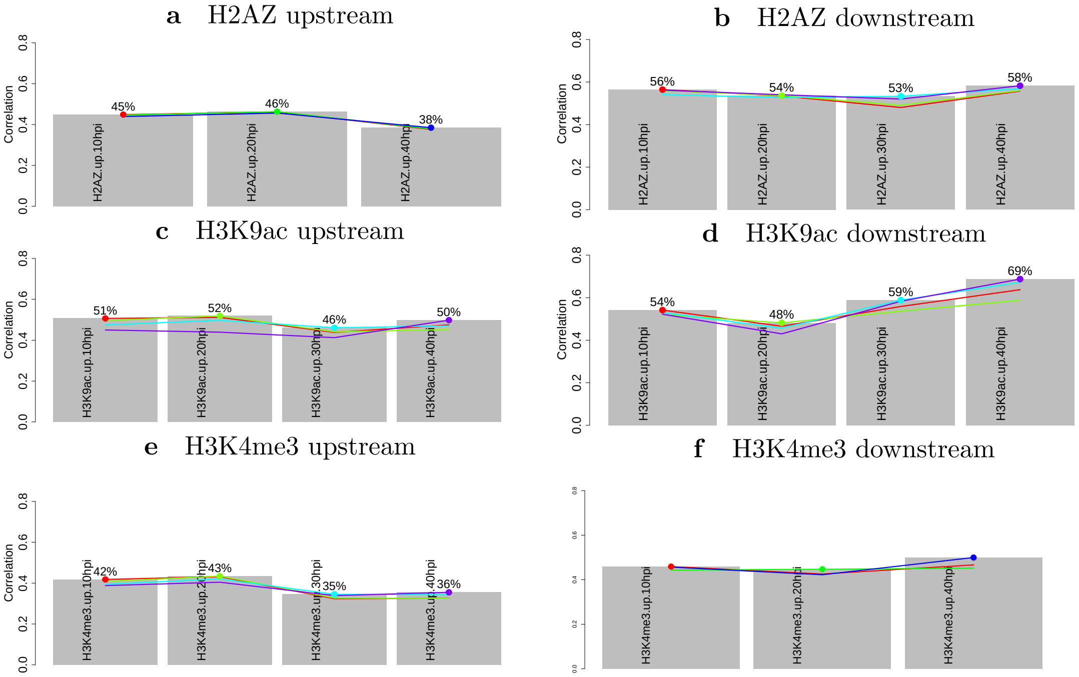

Figure S9: **DExTER accuracy for predicting H2AZ, H3K9ac and H3K4me3 histone marks.** Grey charts represent the accuracy, measured as the correlation between predicted and observed histone mark signal, on 4 time-points. Colored curves summarize the accuracy of a model learned on a specific time point when used to predict other time points of the same series.
