# Supplementary material for: Identification of long regulatory elements in the genome of Plasmodium falciparum and other eukaryotes
Source: PLoS Comput Biol. 2021 Apr 16;17(4):e1008909. doi: 10.1371/journal.pcbi.1008909 (PMC8081344; doi:10.1371/journal.pcbi.1008909)
Supplement: S10 Fig — (PDF) [file pcbi.1008909.s010.pdf]

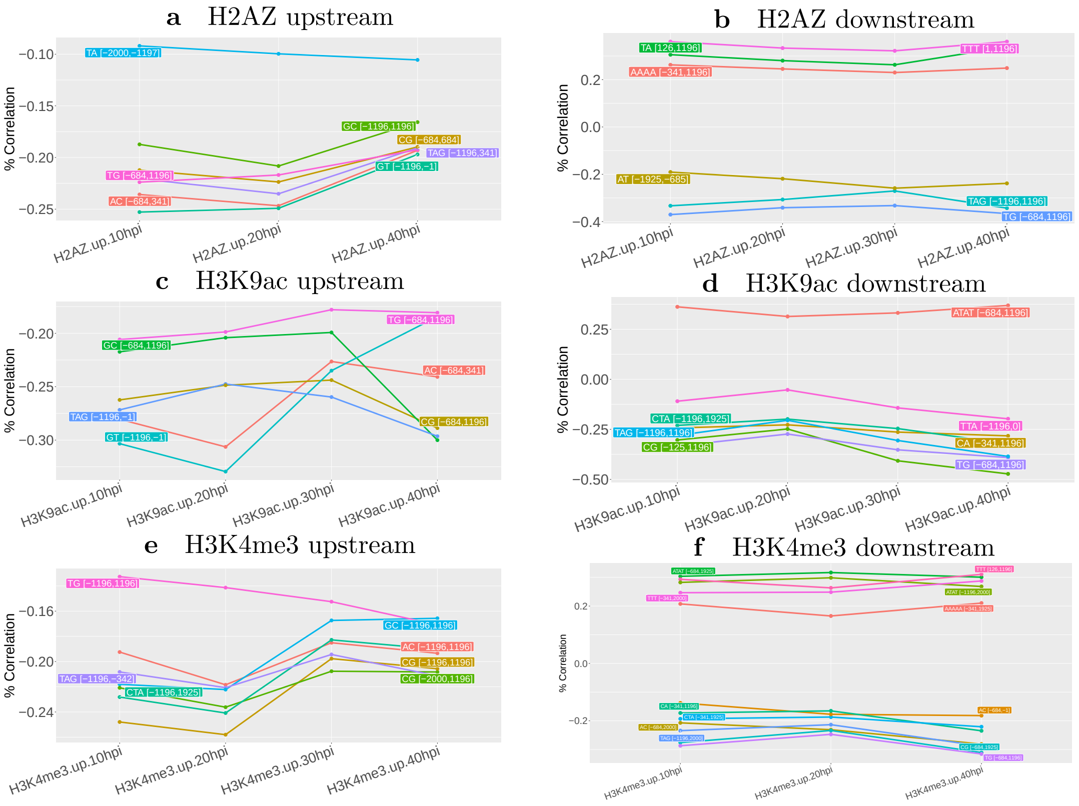

Figure S10: **Correlations between histone mark signal and k-mer frequency of the most important variables identified for the different histone marks and time points.** For each histone mark signal (upstream or downstream AUG), the 10 most important variables of each time points were identified, and their correlations to the histone mark were computed for all time points of the series.
