# Supplementary material for: Identification of long regulatory elements in the genome of Plasmodium falciparum and other eukaryotes
Source: PLoS Comput Biol. 2021 Apr 16;17(4):e1008909. doi: 10.1371/journal.pcbi.1008909 (PMC8081344; doi:10.1371/journal.pcbi.1008909)
Supplement: S11 Fig — (PDF) [file pcbi.1008909.s011.pdf]

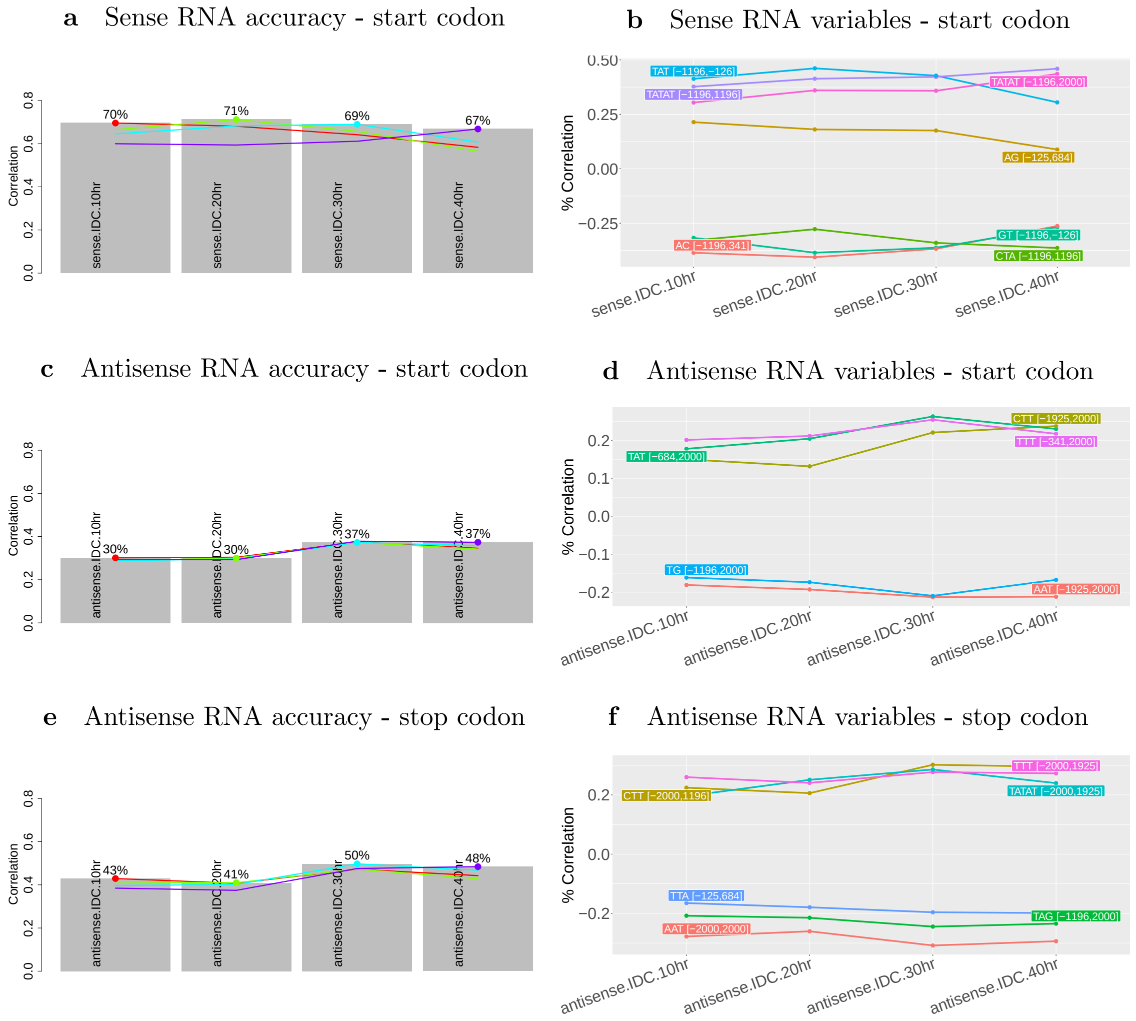

Figure S11: **Prediction of sense and antisense transcript levels.** **(a, b)** Accuracy and best variables of models learnt on sense RNA level using 4000bp sequences centered on start (AUG) codons. **(c, d)** Accuracy and best variables of models learnt on antisense RNA level using 4000bp sequences centered on start codon. **(e, f)** Accuracy and best variables of models learnt on antisense RNA level using 4000bp sequences centered on stop codons. Plots a, d and e, represent the accuracy (grey charts), measured as the correlation between predicted and observed histone mark signal, on 4 time-points. Colored curves summarize the accuracy of a model learned on a specific time point when used to predict other time points of the same series. Plots b, d and f represent the correlations between gene expression and the most important variables identified for each conditions.
