# Supplementary material for: Identification of long regulatory elements in the genome of Plasmodium falciparum and other eukaryotes
Source: PLoS Comput Biol. 2021 Apr 16;17(4):e1008909. doi: 10.1371/journal.pcbi.1008909 (PMC8081344; doi:10.1371/journal.pcbi.1008909)
Supplement: S12 Fig — (PDF) [file pcbi.1008909.s012.pdf]

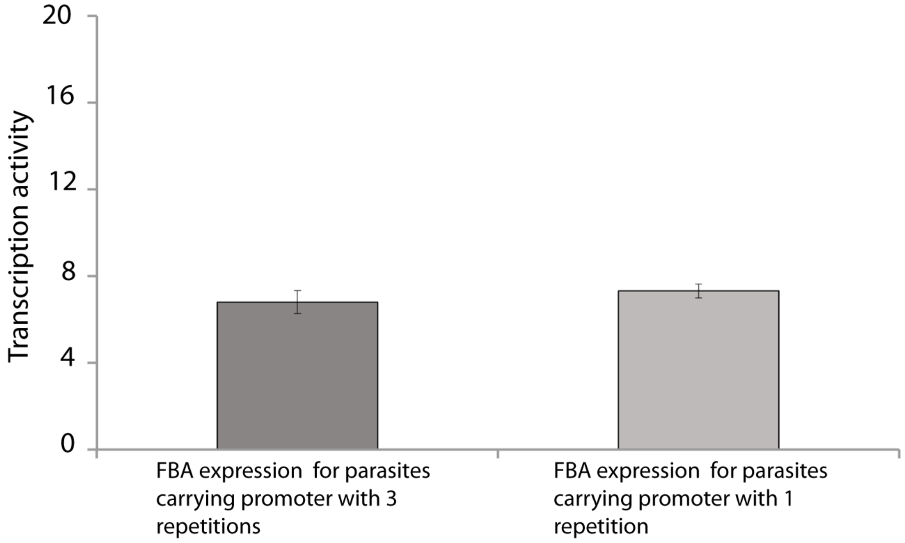

Figure S12: **Control of transcriptional activity of one housekeeping gene in the two genetically modified parasites.** Transcriptional activity quatification by qPCR analysis of RNA collected at ring stages parasites for the gene FBA (PF3D7_1444800) in both transgenic lines.
