# Supplementary material for: Identification of long regulatory elements in the genome of Plasmodium falciparum and other eukaryotes
Source: PLoS Comput Biol. 2021 Apr 16;17(4):e1008909. doi: 10.1371/journal.pcbi.1008909 (PMC8081344; doi:10.1371/journal.pcbi.1008909)
Supplement: Attachment — Submitted filename: review.responses.pdf [file pcbi.1008909.s014.pdf]

Identification of long regulatory elements in the genome of Plasmodium falciparum and other eukaryotes

Dear Editors,

We are pleased to submit a revised version of our paper (manuscript PCOMPBIOL-D-20-01622).

Following your recommendation, we provide here a new version that addresses all points raised by referees. Below is a point-by-point response to all concerns, specifying where in the manuscript the changes have been made.

We would like to thank the three referee who have, by their comments and suggestions, contributed to improve the manuscript.

We hope that our manuscript will now be acceptable for publication in PLOS Computational Biology and look forward to hearing from you.

Sincerely,

Laurent Bréhélin

Reviewer #1:

```
Dear editor, The author Menichelli et al has reported a method to
 identify long regulatory elements in Plasmodium falciparum and some
 other eukaryotes. The paper is of interest to the research community
 of apicomplexan research. I recommend the authors compile their
 findings, and give concrete numbers of LRE identified and make them
 easily accessible for other. Additionally, however, I have the
 following remarks and questions:

1.DexTER, the acronym for the method, was not defined until much later
 in the manuscript. A brief description of one or two sentences, of
```

the method should be mentioned in the abstract.

Thank you for the kind comments. An explanation of the DExTER acronym has been added in the abstract.

2."LERs appear to determine a very large part of gene expression variation", page 2. This is not precise language for reporting science. Can the authors define " a very large part"

Done, see abstract.

3."For most specie, we show that these LRE are predictive of expression with an accuracy in between 50% and 60%, for P. falciparum, the accuracy even exceeds 70%", page 5, these numbers 50%, 60% 70%, do not have a context. Please provide bench marking and rationale for the accuracy, because these number will determine the quality of the research

4.Related to 3, also page 7&8, please provide a benchmark comparison, how much can be recovered, which category, of LERs with at least one other species, e.g. human.

We agree that these statistics should be contextualized and compared to some kind of baseline predictors for assessing their importance. We thus added several experiments to provide benchmark comparison. Notably, we assessed the accuracy of simpler models that only use the frequencies of the 16 dinucleotides computed on the whole 4000bp sequences (Figure S1 and text p. 9) . The accuracy of these models drops, which illustrates the importance of higher level k-mers and of a proper definition of the region boundaries. Also, we compared the accuracy of our approach to that of a predictor that uses TF motif scores instead of the DExTER variables (Figure S2 and text p.9 and 10), and we show that the accuracy of these models is far from that obtained with DExTER variables.

For P. falciparum, these results can also be compared to the accuracy of the convolutional neural networks (CNN) that we trained on the same data (see Figure S8 and text p. 11). Although CNNs are much more sophisticated than the linear models used in DExTER (with a number of parameters substantially higher) in these experiments they do not show better accuracy than DExTER. This point is more largely discussed in section Discussion (p. 21-22).

5."We took 4kb centered either around the gene start, or around AUG ..", page 6, please provide rationale for choosing 4000 bp.

We took +-2kb around the TSS or AUG because for P. falciparum (which constitutes the main targets of this work) and for several other organisms of this study (P. berghei, T. gondii, S. cervevisiae, D. discoidium) the median intergenic region is around 2kb or less (see Table 2 in the paper https://bmcgenomics.biomedcentral.com/articles/10.1186/1471-2164-14-267). This point has been added in Methods.

6.Is there a supplemental file with all the LERs characterized? So that other researchers can perform independent validations. And the author's work will be more accessible for the community.

We added an archive containing all LREs identified for all species in the form of a spreadsheet file. In this tabular, genes are in rows, and LREs in columns. In this way, users can easily rank the genes with respect to a chosen LRE and identify the genes for which the k-mer frequency is the highest/lowest in the identified region. See file "best.features.zip" in Appendix.

7. Fig 5, why is P. falciparum's result so different than that of P. berghei?

This is an interesting remark. Actually a large part of the differences comes from the fact that LREs localized in distant promoter regions seem less important in P. berghei than in P. falciparum. In P. falciparum, a close look at variable importance (Figure 4 and S6) shows that the importance of this region is mostly brought by the frequency of TAT (or ATA), which is highly correlated with expression in the early time points of the intraerythrocytic cycle (and which is btw the variable we experimentally assessed by CRISPR/Cas9). Although this variable is also correlated with expression in P. berghei (the correlation is above 20% for most time points), it is not among the best predictive variables for this organism. This point may be linked to the fact that, although the AT content of the genes of P. berghei and P. falciparum are almost the same (76.22% and 76.25%, respectively), the AT content of intergenic regions is more skewed in P. falciparum (85.72% vs. 80.05%), see https://www.ncbi.nlm.nih.gov/pmc/articles/PMC5857377/ This point has been added in the Discussion.

Reviewer #2:

Summary comments: Overall this is an interesting manuscript that explores the association of long repeat elements (LRE) with gene expression in a number of organisms. The main focus of the work is developing a systematic method to discover LREs adjacent to known or predicted genes and then to assess the association of those sequences with gene expression profiles. For most organisms, the association fluctuates around 60%, but in the case of Plasmodium falciparum is higher and this manuscript focuses on the implications for P. falciparum gene expression. In P. falciparum, whose genome is known to be strongly depleted of transcription factors, LREs appear to determine a very large part of gene expression variation, and their analyses show that they are involved in both transcriptomic and post-transcriptomic regulation mechanisms.

This work is novel and does provide an interesting set of correlative data for the potential role of long repeat elements in the regulation of P. falciparum gene expression. This is quite intriguing because there remain major questions as to the mechanism of gene regulation. There is a very structured program of gene regulation throughout the life cycle and a paucity of predicted transcriptional regulatory proteins.

While the work presented here is of potential interest, a major question as to the relevance of these findings remains. The Plasmodium falciparum genome has an unbalanced base composition with 82% A+T on average across the genome. Most of the G+C pairs occur in genic regions and parts of the non-genic genome reach nearly 100% A+T with long stretches of homopolymer and low complexity (AT repeats) sequence surround genes. The comparisons to other organisms did not include organisms with similar high A+T content. This could influence the results. Of particular concern is the identified key LREs are TATAT, TTT, TATATA and AA all seem to show strong correlations.

Thank you for these kind comments and for this suggestion.
To assess the link between genome AT content and the predictive performance of LREs, the new version of this manuscript includes the analysis of two supplementary organisms selected for their high genome AT-content (Dictyostelium discoideum et Tetrahymena thermophila). We observed very

different results for these two organisms. On Dictyostelium discoideum, the accuracy achieved by DExTER is close to that obtained on Plasmodium species, and a dynamic behavior is also observed, with marked differences between models learned at the beginning and end of the development course. On the contrary, for Tetrahymena thermophila the accuracy stays around 50% and no dynamic behavior is observed. In conclusion, it is true that the best predictive performances of this study have been obtained with high AT-content genomes (P. falciparum, P. berghei and Dictyostelium discoideum), but it seems that there are also AT-biased genomes for which the link between LREs and gene expression is not as strong. This also indicates that DExTER prediction power is not particularly biased towards AT-rich genomes.These analyses have been included in a new section (see p. 19).

`On the positive side, the authors do show strong associations with particular sequences with certain biological features of gene expression in the parasite and the computational model may be teasing out relationships not previously recognized.`

`The authors extend their analysis to a functional test of the LREs by constructing two chimeric upstream regions, one with high ATA content and one with low ATA content attached to a GFP reporter and then inserting these in the Plasmodium pfs47 gene locus and measuring GFP expression. They observed a 10-fold difference in expression and while potentially interesting probably will require some additional characterization. Pfs47 is sexual stage specifically expressed in its native configuration so the CRISPR/Cas9 inserted construct has in some way changed the native expression profile { perhaps through the disruption of chromatin structure. Before definitive conclusions can be drawn, including additional work to determine if this difference in expression is context or sequence dependent.`

We thank the reviewer for these comments. One of the most important variables identified by DExTER is the ATA frequency in region [-1196,-126]. We have built a chimeric promoter containing 3 repetitions of a region with high ATA frequency (Fig 10). As a control, we constructed a second promoter replacing two of these repetitions by a region of low ATA frequency. Both of these chimeric promoters were of similar length and GC content. These expressing cassettes were inserted into the locus Pfs47, shown to be dispensable for parasite blood stage development and previously used for transgene expression of GFP and other genes. (Knuepfer et al., 2017; Talman et al., 2020). The different results obtained with the two different sequences in the

same locus indicate that the differences are sequence dependent and not context dependent. These results are explained in the Result section, in Fig. 10 and commented in the Discussion.

It would be particularly interesting of insertion of a specific LRE disrupted chromatin structure.

We agree with the referee that it would be interesting but we feel that it is beyond the scope of this study.

Overall, this work is of potential interest and may shed light on the mechanisms associated with the control of gene expression. The work is innovative and novel and has opened some new questions that merit further experimental exploration.

Thank you very much for these comments.

Reviewer #3:

In the manuscript entitled "Identification of long regulatory elements in the genome of Plasmodium falciparum and other eukaryotes" by Menichelli et al., authors have used a computational approach to identify the long regulatory elements (LREs) in the genomes of various eukaryotic systems. They have also used various conditions to train the program to eventually use it to identify the LREs and predict the gene expression from unknown sequences. Authors have used expression data as a readout for the direct association of the long regulatory elements with the regulation of the associated genes. Finally, they have validated the association between the LREs and gene expression in Plasmodium falciparum using two LREs of varying length in in vitro conditions.

In this study, investigators have addressed an important long-standing question in the field and identified novel LREs and their association with gene expression. While the analysis is rigorous and potentially very interesting the manuscript needs clarity in presentation and explanation. Here are some of the concerns outlined.

1. One of the major concerns is that authors have used the association between LREs and gene expression as causation (i.e. regulation) interchangeably throughout the manuscript. Though causation is

possible, the data provided in this manuscript is not sufficient to establish the causal relationship.

We fully agree with this concern. In this new version of the manuscript we have rephrased all sentences that were implying a direct causation link between gene expression and LREs. Also, to avoid any mistake, the term LRE (Long Regulatory Element) has been replaced by cLRE (candidate Long Regulatory Element) in most places. We kept the term LRE only for general sentences that do not refer to a particular element, and, at the end of the manuscript, for the upstream ATA element that has been tested by reporter assay.

2. The correlation (which I believe is Pearson correlation) between LREs and expression is generally in the range of 0.2 to 0.5 in Figure 4. These are mostly weak or moderate associations.

It is true that the correlations between cLREs and gene expression are often moderate. However this was something expected if we consider that the measured gene expressions level is actually the results of several regulatory mechanisms. This point is now discussed in the paper (p. 12).

3. Most of the housekeeping genes in higher eukaryotic systems (human and mouse) have high CpG content at promoters. Thus sequence itself is governing the gene expression in this case. How do the authors explain the sinusoidal pattern of gene expression in Plasmodium with LREs?

We acknowledge that the dynamic behavior observed in Plasmodium and other species may appear somewhat surprising as it is dictated by the sequence of the genome, which is intrinsically static. This is however simply the reflect of the interplay that occurs in cells between cis-elements—which are statics—and DNA-binding molecules, whose nature and quantity vary depending on conditions. Thus, the presence of a specific cis-element in the vicinity of certain genes may for example induce high expression in conditions where the associated DNA-binding molecule is active, while being useless in other conditions. In our framework, this is translated by the fact that the models learned at different conditions do not use the same variables (cis-elements). Hence, a model learnt at a given condition may have poor accuracy on other conditions. This comment has been added in the Discussion (p. 21). Note also that we are not the first authors to propose to learn condition-specific models (see for example https://pubmed.ncbi.nlm.nih.gov/32433972/).

See also https://pubmed.ncbi.nlm.nih.gov/33134611/ for interesting discussion about this point, and others.

4. I was wondering if DExTER has also identified CpG elements in the human/mouse genome? Does it correlate with gene expression?

Yes, the frequency of dinucleotide CG in region around the TSS is indeed the most predictive variable in human, both in tissues and developmental series. This remark has been added in the manuscript (see p.12).

5. The design for in vitro experimental validation is not clear. In this experiment, the authors have used two different sequences and compared the GFP expression. As these are two different sequences they cannot be directly compared. Authors should take varying repetitions of the ATA sequences (eg. 3, 2, and 1) to see the effect of this LTR on expression. Moreover, if they want to demonstrate this association for causation, they need to mutate the LRE of the same length.

We thank the reviewer for their comment. We have better explained the design of the experiment (see Results and Methods) and also changed the Fig. 10. One of the most important variables identified by DExTER is the ATA frequency in region [-1196,-126]. Therefore, we have built a chimeric promoter containing 3 repetitions of a region with high ATA frequency. As a control, we constructed a second promoter replacing two of these repetitions by a region of low ATA frequency. Both of these chimeric promoters were of similar length and GC content. These expressing cassettes were inserted into the locus Pfs47, shown to be dispensable for parasite blood stage development and previously used for transgene expression of GFP and other genes. (Knuepfer et al., 2017; Talman et al., 2020). The different results obtained with the two different sequences in the same locus indicate that the differences are sequence dependent and not context dependent.

6. It would be important that authors provide information pertinent to the important LREs identified in a tabulated form. LRE-sequence/associated with number of genes/LRE count/gene ontology/significance value.

As asked by Referee 2 also, we added an archive containing all LREs identified for all species in the form of a spreadsheet file. In this tabular, genes are

in rows, and LREs in columns. In this way, users can easily rank the genes with respect to a chosen LRE and identify the genes for which the k-mer frequency is the highest/lowest in the identified region.

7. Which version of the Plasmodium genome is used for the analysis?

We used the 3D7 genome version Jun 18, 2015, downloaded from PlasmoDB. The versions of the different genomes used in these analyses have been added in Material and Methods (p. 31-32).

8. Authors have also looked at conservation of the identified LREs along evolution. Why do authors believe that there will be conservation in LREs when genomes are so different? Please include an explanation.

We did not really expect to observe conservation at such very high evolutionary distance. The idea was rather to check if some elements could be conserved between the closest species of this study (Plasmodiums, Apicomplexa, and may be the Ecdysozoa group whose insects and worms belong to). Actually, several studies have shown that regulatory elements are sometimes conserved at high phylogenetic distance. For example, the role of CpG islands in transcription regulation is likely shared at the vertebrate level (https://www.ncbi.nlm.nih.gov/pmc/articles/PMC3093116/). Also, a recent study suggests that the enhancer regulatory code may be conserved at the animal level (https://science.sciencemag.org/content/370/6517/eaax8137). This point has been added in the manuscript (see p.13).

9. The text in figure 1 is very hard to read due to the very small font size.

This point has been modified.

10. Axis label is missing in figure 2. Also, tissue type/stages of development are not explained properly in the figure or the text.

Axes labels have been added. Names of tissues and development stages have been clarified when necessary.

11. Figure 4: The ‘most important variables’ indicated on the graph are hard to read due to the poor contrast and small font size.

This point has been modified.

12. In general, the font sizes of the axis labels are too small for many supplementary figures. Figure caption is missing for figure S3. Axis label is missing in Figure 7a, S5 and S9.

These points have been modified.

13. Authors should specify moderate/ average/higher gene expression correlations.

We agree that these terms are vague and not scientifically sound, so we tried to avoid them in this new version of the paper.

14. The assumption that LREs are involved in post-transcriptional regulation is not supported in this study. Plasmodium also produces a lot of anti-sense RNAs and these LREs might have a role in transcriptional regulation of anti-sense RNAs.

The question of the role of LRE in post-transcriptional regulation is addressed in the paper. Notably, we analysed the data of Painter et al. (2018), who measured separately nascent and stabilized RNA, and we see (Figure 8b) that certain cLREs are more correlated with transcription data, while others are more correlated with stabilization data. The name of this Figure has been changed for clarification.

Concerning the role cLREs may have on transcriptional regulation of antisense RNA, we included in the new manuscrit an analysis of the data of Siegel et al. (2014) who measured separately the level of sense and antisense RNA of coding genes. When predicting these data with DExTER, we observed that the accuracy achieved for antisense RNA was significantly lower than that of sense RNAs. Different reasons may explain this discrepancy but one possibility would be that the very low expression level of antisenses actually hinders their proper quantification. Interestingly however, we obtained better results on antisense RNAs when DExTER uses genomic sequences centered on the stop codon rather than on the start codon for the predictions. This suggests that these transcripts may possess their own promoter sequences, and that LREs can be also involved in their regulation indeed (see p. 18).
