# Supplementary material for: Identification of long regulatory elements in the genome of Plasmodium falciparum and other eukaryotes
Source: PLoS Comput Biol. 2021 Apr 16;17(4):e1008909. doi: 10.1371/journal.pcbi.1008909 (PMC8081344; doi:10.1371/journal.pcbi.1008909)
Supplement: Attachment — Submitted filename: review.responses.210224.pdf [file pcbi.1008909.s015.pdf]

Identification of long regulatory elements in the genome of Plasmodium falciparum and other eukaryotes

Dear Editors,

We are pleased to submit a revised version of our paper (manuscript PCOMP-BIOL-D-20-01622).

Following your recommendation, we provide here a new version that addresses the points raised by referee #2, along with additional analyses. Note that our wet lab experiments follow very classical procedures, that are widely used in the field of *Plasmodium* molecular biology. We are ready to provide more references using similar procedures to substantiate our responses if necessary. Below is a point-by-point response to all concerns, specifying where in the manuscript the changes have been made.

We hope that our manuscript will now be acceptable for publication in PLOS Computational Biology and look forward to hearing from you.

Sincerely,

Laurent Bréhélin

Reviewer #1:

```
Most of my questions have been answered or addressed.
```

Reviewer #2:

```
The authors have responded with new data and a modified text to the
 comments by the referees. The addition of the analysis of other high
 A+T content organisms provides additional new data and strengthens
 the manuscript. There remains one issue that was not adequately
```

addressed. The functional validation remains a weak point in the manuscript. The authors have not addressed issues of genomic context - in particular, the authors have used a gene that is normally only expressed in the mosquito stages and yet they measure the transcription in the asexual stages.

We thank the reviewer for their comment. As explained in the results section, we used to validate our approach a part of the promoter of the PF3D7_0913900 gene (only the promoter, not the gene itself). This gene is expressed in asexual stages, exactly the stage in which the experiment has been done. We have higlighted this in the results section (see line 460). Our two chimeric promoters were inserted in a genomic locus that has been previously used for transgene expression in asexual stages of GFP and other genes (see for example references 37 and 82 in the manuscript). This has been as well highlighted in the text (see line 473).

There are no controls presented to demonstrate that transcriptional levels of other genes have similar transcriptional levels in the two genetically modified parasites.

As described in Methods, the quantitative PCR was done by normalizing expression of our constructs by those of two housekeeping genes (seryl-tRNA synthetase PF3D7_0717700, and fructose-biphosphate aldolase PF3D7_1444800, see lines 874-875). This is the standard way to control for such potential effect. For the reviewer purpose, below is an additional Figure that shows that the transcriptional activity of the FBA gene is similar in the two genetically modified parasites:

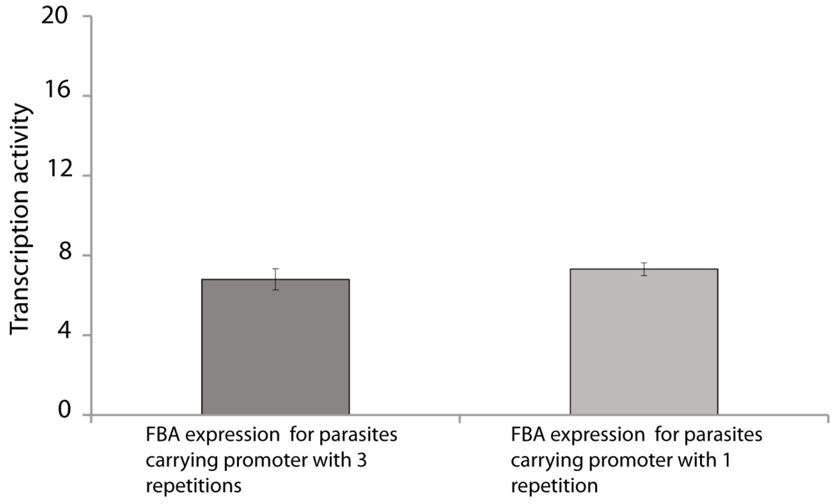

Transcriptional activity quatification by qPCR analysis of RNA collected at ring stages parasites for the gene FBA (PF3D7_1444800) in both transgenic lines.

```
There is not confirmation that the two lines are assayed
 in parasites at the same stage of the life cycle. Without additional
 experiments including key control experiments, the data presented
 cannot be used as proof of validation. This is the beginning of a
 validation process, but far from the acceptable standard.
```

As described in Methods, the two transgenic lines were synchronized (purification + sorbitol treatment) and both were collected at 14-hours post-invasion (lines 863-866). This has been confirmed by microscopy (Giemsa staining). We have edited the text in material and methods to gain in clarity about this question (see line 866).

Reviewer #3:

```
In th manuscript entitled "Identification of long regulatory elements
 in the genome of Plasmodium falciparum and other eukaryotes" by
 Menichelli et al., Authors have satisfactorily answered all my
 concerns.
```
