# Supplementary material for: Identification of long regulatory elements in the genome of Plasmodium falciparum and other eukaryotes
Source: PLoS Comput Biol. 2021 Apr 16;17(4):e1008909. doi: 10.1371/journal.pcbi.1008909 (PMC8081344; doi:10.1371/journal.pcbi.1008909)
Supplement: Attachment — Submitted filename: review.responses.210317.pdf [file pcbi.1008909.s016.pdf]

Identification of long regulatory elements in the genome of Plasmodium falciparum and other eukaryotes

Dear Editors,

We are pleased to submit a revised version of our paper (manuscript PCOMPBIOL-D-20-01622). We provide here a new version that addresses your and the reviewer's recommendations.

Specifically, we have now included the data presented only for the reviewer in supplemental material (see lines 478-481 and Figure S12).

We have also modified the last sentence of the Abstract, replacing the term "demonstrated" by "assessed" which is much less conclusive. We think however that it is important to keep mention of our experimental validation in the abstract. While we agree with the reviewer that additional experiments are required to further investigate the role and importance of LREs in *Plasmodium* biology (this is now discussed in the manuscript, see lines 543-548), we also think that our in vivo experiment with two chimeric promoters constitutes a first and promising attempt toward this goal.

As requested, lines 478-479 ("These results validate DExTER for identifying LREs, and demonstrate the importance 478 of these elements for the control of gene expression in P. falciparum.") have been deleted.

We hope that our manuscript will now be acceptable for publication in PLOS Computational Biology and look forward to hearing from you.

Sincerely,

Laurent Bréhélin, Charles Lecellier and Jose-Juan Lopez-Rubio
